# From Euphoria to Cardiac Stress: Role of Oxidative Stress on the Cardiotoxicity of Methylone and 3,4-DMMC

**DOI:** 10.3390/toxics13110998

**Published:** 2025-11-20

**Authors:** Maria Moreira, Verónica Rocha, Ana Margarida Araújo, Márcia Carvalho

**Affiliations:** 1 Instituto de Investigação, Inovação e Desenvolvimento Fernando Pessoa (FP-I3ID), Fernando Pessoa University, Fernando Pessoa Teaching and Culture Foundation, Praça de 9 de Abril 349, 4249-004 Porto, Portugal; 41114@ufp.edu.pt (M.M.); 41143@ufp.edu.pt (V.R.); 2 Laboratório Associado para a Química Verde/Rede de Química e Tecnologia (LAQV/REQUIMTE), Laboratory of Bromatology and Hydrology, Department of Chemical Sciences, Faculty of Pharmacy, University of Porto, 4050-313 Porto, Portugal; 3 RISE-Health, Faculty of Health Sciences, Fernando Pessoa University, Fernando Pessoa Teaching and Culture Foundation, Rua Carlos da Maia 296, 4200-150 Porto, Portugal

**Keywords:** synthetic cathinones, cardiotoxicity, H9c2 cells, oxidative stress, antioxidants

## Abstract

Synthetic cathinones (SCs), commonly referred to as “bath salts”, are a class of novel psychoactive substances (NPSs) that elicit amphetamine-like effects and severe cardiovascular outcomes, including myocardial infarction and sudden cardiac death. Despite these risks, the mechanisms underlying SC-induced cardiotoxicity remain poorly studied. This study investigated the in vitro cardiotoxicity of two prevalent SCs—methylone and 3,4-dimethylmethcathinone (3,4-DMMC)—in H9c2 rat cardiomyoblasts, focusing on oxidative stress and the potential protective role of antioxidants. Cells were exposed to methylone (0.01–4.0 mM) or 3,4-DMMC (0.0005–0.8 mM) for 24 and 48 h, and cytotoxicity was assessed by an MTT assay. Intracellular reactive oxygen/nitrogen species (ROS/RNS) were quantified by fluorescence, and antioxidant effects were evaluated using ascorbic acid, N-acetylcysteine, and Trolox. Both SCs caused concentration-dependent cytotoxicity, with 3,4-DMMC showing higher potency than methylone (IC_50_: 0.28 vs. 0.98 mM, *p* = 0.0013). ROS/RNS levels increased in a concentration- and time-dependent manner for both compounds, reflecting early and sustained redox imbalance. Of the antioxidants, only ascorbic acid significantly improved cell viability. Taken together, these findings demonstrate for the first time that methylone and 3,4-DMMC exert cardiotoxic effects in vitro, with oxidative stress as a key contributor. The protective effect of ascorbic acid highlights its potential as a therapeutic candidate against SC-induced cardiac injury.

## 1. Introduction

New psychoactive substances (NPSs) continue to pose a global public health challenge due to their rapid emergence, chemical diversity, and widespread availability [1,2]. Designed to mimic the psychoactive effects of controlled drugs such as cannabis, cocaine, and amphetamines while circumventing drug legislation, NPSs remain attractive to recreational users [3]. According to the European Drug Agency (EUDA) 2025 report, although the rate of appearance of new compounds has slowed since 2015, overall demand and availability remain high, fueled in large part by online distribution channels. Among these substances, synthetic cathinones (SCs) consistently rank as the second most frequently seized group of NPSs, surpassed only by synthetic cannabinoids [4].

SCs are β-keto analogues of amphetamines, structurally related to cathinone, the active alkaloid in the khat plant (*Catha edulis*) [2,5]. Commercially marketed as “bath salts”, “plant food”, or “research chemicals”, SCs are frequently mislabeled, facilitating widespread misuse. They became popular on the illicit market due to their amphetamine-like psychostimulant effects, which are commonly associated with increased energy and motivation, euphoria, enhanced sociability, and intensified sexual drive [2,6].

Among the many SCs, two derivatives have gained particular attention: 3,4-methylenedioxy-N-methylcathinone (methylone) and 3,4-dimethylmethcathinone (3,4-DMMC) (structures shown in Supplementary Figure S1). Both are often described as “MDMA-like” drugs, due to their stimulant and empathogenic properties [7]. Methylone acts as a non-selective monoamine releaser and reuptake inhibitor, elevating extracellular levels of dopamine, serotonin, and norepinephrine [8]. In contrast, 3,4-DMMC functions primarily as a potent transporter substrate, with higher selectivity for serotonin and norepinephrine transporters [9]. However, beyond their desired effects, SC use has been linked to serious toxic outcomes across multiple organ systems [2] and implicated in numerous fatal intoxications [10,11,12].

The cardiovascular system is one of the primary targets of SC-related toxicity. Clinical and forensic reports document tachycardia, hypertension, chest pain, arrhythmias, QT prolongation, myocardial infarction, and sudden cardiac death—even in young individuals without preexisting conditions [10,13,14,15]. These events are believed to arise from synergistic mechanisms, including indirect effects of excessive sympathetic nervous system stimulation [14] and direct cardiomyocyte effects such as ion channel interference and disruption of electrophysiological stability [16,17]. Mitochondrial dysfunction and oxidative stress have also been implicated as central contributors to SC toxicity [15,18], consistent with mechanisms described for amphetamine-type stimulants [19,20]. Nonetheless, the specific role of oxidative stress in mediating SC-induced cardiotoxicity remains poorly defined, representing an important knowledge gap with implications for both toxicological risk assessment and the development of targeted interventions.

In this study, we investigated the in vitro cardiotoxic potential of methylone and 3,4-DMMC using the H9c2 rat cardiomyoblast cell line, a well-established model for cardiotoxicity research [21]. Our objectives were to: (i) characterize concentration- and time-dependent effects on cell viability; (ii) assess intracellular reactive oxygen/nitrogen species (ROS/RNS) generation and redox imbalance; and (iii) evaluate the potential protective role of the antioxidants ascorbic acid, N-acetylcysteine, and Trolox. By focusing on oxidative stress as the key mechanistic pathway, we aim to clarify the cardiac toxicity of these two prevalent SCs and to explore potential avenues for protective intervention.

## 2. Materials and Methods

### 2.1. Chemicals

All reagents used in this study were of analytical grade and sourced from certified suppliers. Dulbecco’s Modified Eagle Medium (DMEM), fetal bovine serum (FBS), penicillin-streptomycin (10,000 U/mL/10,000 μg/mL), and dimethyl sulfoxide (DMSO) were obtained from PAN Biotech (Aidenbach, Germany). N-acetylcysteine (NAC), ascorbic acid (AA), Trolox, 3-(4,5-dimethylthiazol-2-yl)-2,5-diphenyltetrazolium bromide (MTT), and Triton X-100 were purchased from Sigma-Aldrich (St. Louis, MO, USA). Trypsin was acquired from Biowest (Nuaillé, France). Methylone and 3,4-DMMC were obtained from Sensearomatic website and were fully characterized by mass spectrometry and elemental analysis, presenting a confirmed purity greater than 98%.

### 2.2. Cell Culture and Experimental Design

The H9c2 cell line (ATCC CRL-1446), derived from embryonic rat ventricular tissue, is widely employed in cardiovascular research due to its preserved cardiac phenotype and functional properties [22]. Cells were cultured in DMEM supplemented with 10% FBS, 1% penicillin/streptomycin, and maintained in a humidified incubator at 37 °C and 5% CO_2_. Subcultures were performed weekly by trypsinization, and the assays were conducted within 10 passages (passages 9–18) to minimize phenotypic changes. As this is an established cell line, no animal experimentation was performed, and the study was conducted in accordance with international ethical standards for research integrity.

For toxicological assays, H9c2 cells were seeded at a density of 1 × 10^4^ cells per well in 48-well culture plates. Drug treatments were conducted in DMEM supplemented with 1% FBS to minimize nonspecific binding of cathinones to serum proteins. To generate complete cytotoxicity concentration–response curves, ranging from no detectable cytotoxicity to complete cell death, H9c2 cells were exposed to methylone or 3,4-DMMC for 24 or 48 h at concentration ranges of 0.01–4.0 mM and 0.0005–0.8 mM, respectively. Cell viability was assessed indirectly using the MTT reduction assay, which provides an assessment of mitochondrial metabolic activity. IC_10_, IC_40_, and IC_50_ values were calculated from the concentration–response curves.

To assess ROS and RNS production, cells were exposed to the drugs for 0.5, 1, 2, 3, 4, 5, 24, and 48 h at concentrations corresponding to the IC_10_ and IC_50_ values estimated from the 24 h concentration–response curves obtained with the MTT reduction assay. The determined concentrations were as follows: for methylone, IC_10_ = 0.119 mM and IC_50_ = 0.978 mM; for 3,4-DMMC, IC_10_ = 0.079 mM and IC_50_ = 0.280 mM.

For experiments evaluating the potential protective effects of antioxidants on drug-induced cytotoxicity, cells were pretreated for 30 min with 0.1 mM ascorbic acid (AA), 1 mM N-acetylcysteine (NAC), or 0.2 mM Trolox, followed by co-incubation for 24 h with methylone and 3,4-DMMC at their IC_40_ values (0.662 mM for methylone and 0.221 mM for 3,4-DMMC). The IC_40_ was chosen to represent moderate but measurable cytotoxicity, enabling detection of potential antioxidant effects while avoiding excessive and irreversible cellular damage. After treatment, both cell viability and ROS/RNS levels were evaluated under the same experimental conditions.

### 2.3. Cell Viability Assessment

Cell viability following exposure to the test compounds, either individually or in combination with antioxidants, was evaluated using the MTT reduction assay, as previously described [23]. After the exposure period, the culture medium was replaced with MTT solution (0.5 mg/mL), and cells were incubated for 1.5 h at 37 °C. Formazan crystals formed during this process were solubilized in DMSO, and 100 µL of each sample was transferred to a 96-well plate for absorbance measurement at 545 nm with a 630 nm reference using a Varioskan LUX multimode microplate reader (Thermo Scientific, Waltham, MA, USA). Cell viability was expressed relative to untreated controls (negative control) and Triton X-treated cells (positive control). All experiments were performed in three independent experiments, each performed in duplicate.

### 2.4. ROS/RNS Measurement

Intracellular ROS and RNS generation was quantified using the DCFH-DA probe, as previously described [19], with minor modifications. Briefly, the culture medium was removed, and 100 µL of 100 µM DCFH-DA solution was added to each well, followed by incubation at 37 °C in a humidified atmosphere of 5% CO_2_ for 30 min. After incubation, the probe solution was discarded, and cells were exposed to methylone or 3,4-DMMC at concentrations corresponding to IC_10_ and IC_50_. Fluorescence intensity was measured at 0.5, 1, 2, 3, 4, 5, 24, and 48 h on a Synergy HTX multimode plate reader (BioTek, Winooski, VT, USA) with excitation at 485 nm and emission at 530 nm. For experiments assessing antioxidant effects, cells were simultaneously treated with antioxidants and the DCFH-DA probe for 30 min. After removing the solution, cells were exposed to methylone or 3,4-DMMC at their IC_40_ concentrations, and fluorescence intensity was measured after 24 h. Data were obtained from at least three independent experiments performed in duplicate and expressed as fluorescence intensity normalized to untreated controls.

### 2.5. Statistical Analysis

For statistical analysis and construction of non-linear regression models, GraphPad Prism version 10.6.1 was used. Cell mortality curves were expressed as a function of the logarithm of the concentration (mM), based on data obtained from the MTT assay. The model that provided the best fit to the data was the “log(inhibitor) vs. normalized response—variable slope”, described by the following equation: Y = 100/(1 + 10^((LogIC50 − X) × HillSlope)^). In this equation, Y represents the normalized response (cell mortality in %), X the logarithm of the concentration of the compound under study (mM), LogIC50 the logarithm of the concentration that induces 50% cell mortality, and HillSlope the coefficient defining the slope of the curve. Statistical comparison between the fitted curves was performed using the Extra Sum-of-Squares F test.

Data related to antioxidant effects and ROS/RNS production were presented as mean ± standard error of the mean (SEM). Data normality was assessed using the Shapiro–Wilk test. For the antioxidant effect data, differences among experimental conditions were analyzed using one-way ANOVA, followed by Sidak’s multiple comparisons test. For ROS production, a two-way ANOVA followed by Tukey’s multiple comparisons test or a one-way ANOVA followed by Sidak’s multiple comparisons test (for antioxidants co-incubation) were applied.

For all statistical tests, a *p*-value < 0.05 was considered indicative of statistical significance.

## 3. Results

### 3.1. Methylone and 3,4-DMMC Induce Concentration-Dependent, but Time-Independent, Death of H9c2 Cardiac Cells

**Figure 1:**
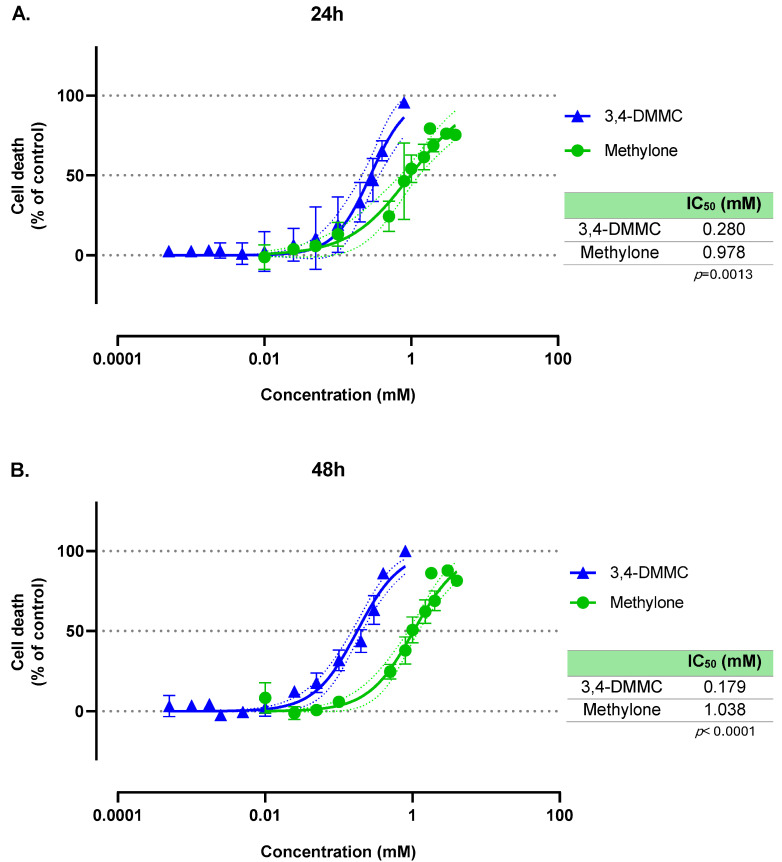
Nonlinear regression models for the cell death induced by methylone (green line) and 3,4-DMMC (blue line) in H9c2 cells, as evaluated by the MTT assay after (**A**) 24 h and (**B**) 48 h of exposure. The mean effects were fitted using a variable-slope nonlinear regression model (log[inhibitor] vs. normalized response). Dotted lines represent the 95% confidence band of each fit. Results were obtained from three independent experiments, performed in duplicate. Embedded tables display the estimated IC_50_ values for each compound at the respective exposure time, as well as *p* value for group comparison (methylone vs. 3,4-DMMC) of global fits.

We first investigated the effects of a broad concentration range of methylone and 3,4-DMMC on the viability of H9c2 cells. Figure 1 shows the concentration-response curves obtained through the MTT reduction assay after 24 h and 48 h of exposure, along with the corresponding IC_50_ values. Both compounds significantly reduced cell viability in a concentration-dependent manner, although with distinct toxic potencies. The cytotoxicity curve for 3,4-DMMC was significantly shifted to the left compared with methylone, reaching statistical significance at 24 h (*p* = 0.0024) and becoming more pronounced at 48 h (*p* < 0.0001). At 24 h, the IC_50_ values were 0.280 mM for 3,4-DMMC and 0.978 mM for methylone (*p* = 0.0013). At 48 h, the IC_50_ values were 0.179 mM for 3,4-DMMC and 1.038 mM for methylone (*p* < 0.0001). These results clearly demonstrate that 3,4-DMMC is more potent than methylone at inducing cytotoxicity.

In contrast, the comparison of the 24 h and 48 h concentration–response profiles for each drug revealed no statistically significant differences (Supplementary Figure S2). This was corroborated by nearly identical IC_50_ values at both time points, indicating that exposure duration had little influence on overall cytotoxicity and that most toxic effects were established within the first 24 h of treatment.

### 3.2. Methylone and 3,4-DMMC Promote ROS and RNS Generation in H9c2 Cardiac Cells

**Figure 2:**
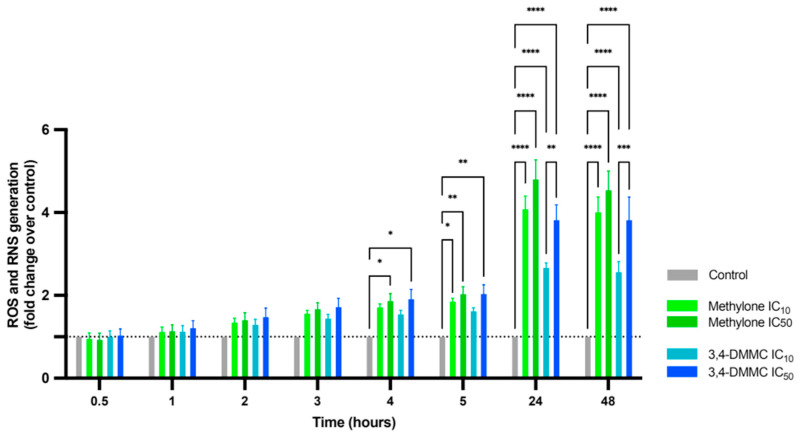
Intracellular ROS and RNS production in H9c2 cells after 30 min, 1 h, 2 h, 3 h, 4 h, 5 h, 24 h and 48 h of exposure to IC_10_ and IC_50_ 3,4-DMMC (blue bars) or methylone (green bars). Data are presented as fold-increase over control (dotted line = 1). Results were obtained from three independent experiments, performed in duplicate. * *p* < 0.05, ** *p* < 0.01, *** *p* < 0.001, and **** *p* < 0.0001.

We next investigated the role of oxidative stress in the observed cell death. A progressive increase in ROS/RNS production over time in cells exposed to both drugs was observed (Figure 2), with statistically significant differences detected as early as 4 h (*p* < 0.01), reaching maximum levels at 24 h and 48 h (*p* < 0.0001). ROS/RNS levels were also concentration-dependent: cells treated at IC_50_ consistently exhibited higher reactive species levels than those treated at IC_10_, reaching significance in cells exposed to 3,4-DMMC at 24 h (*p* < 0.01) and remaining elevated through 48 h (*p* < 0.001).

These results indicate that exposure to methylone and 3,4-DMMC induces oxidative stress in a time- and concentration-dependent manner, supporting its role as a key mechanism underlying their cardiotoxic effects.

### 3.3. Ascorbic Acid, but Not NAC or Trolox, Prevents Methylone- and 3,4-DMMC-Induced Toxicity

**Figure 3:**
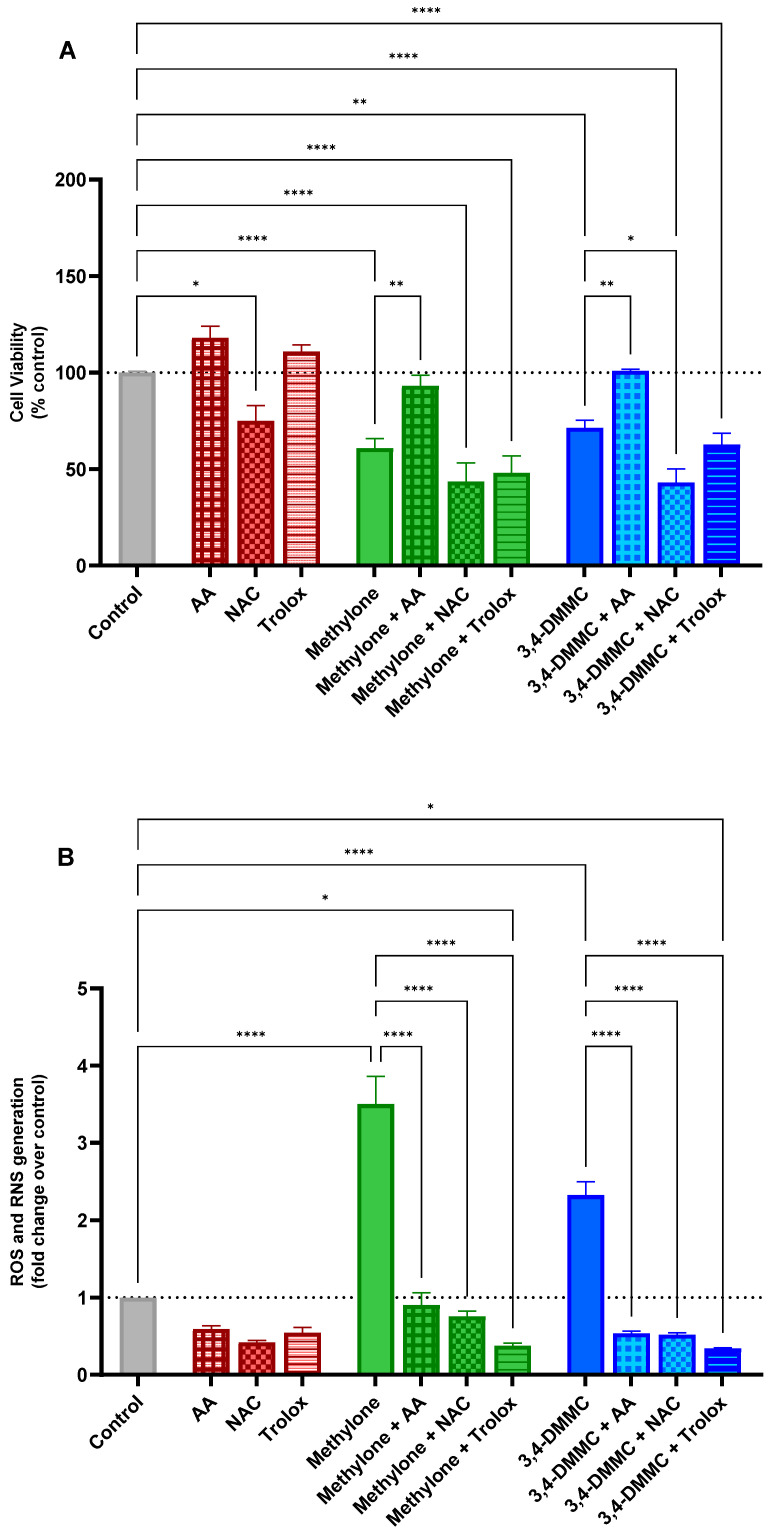
Cell viability (**A**) and intracellular ROS/RNS levels (**B**) of H9c2 cells exposed for 24 h to the IC_40_ of methylone or 3,4-DMMC, in the absence or presence of the antioxidants: ascorbic acid (AA, 0.1 mM), N-acetylcysteine (NAC, 1 mM), or Trolox (0.2 mM). The dashed line represents the control level (set to 100% for viability and 1-fold for ROS/RNS), serving as a reference for comparing treatment effects. Data represent mean ± SEM from at least three independent experiments * *p* < 0.05, ** *p* < 0.01, and **** *p* < 0.0001.

To further evaluate the role of oxidative stress to SC-induced cytotoxicity, we examined the effects of the antioxidants AA, NAC, and Trolox on the viability of H9c2 cells exposed to methylone or 3,4-DMMC at their IC_40_ concentrations for 24 h. As shown in Figure 3A, when tested individually, the antioxidants produced distinct outcomes. At the tested concentrations, AA and Trolox had no significant effects on cell viability compared with untreated controls, whereas NAC alone significantly reduced cell viability to approximately 75% (*p* < 0.05), indicating an intrinsic cytotoxic effect of this antioxidant on H9c2 cells. When cells were pretreated with antioxidants prior to exposure to methylone or 3,4-DMMC, only AA conferred significant protection, fully preserving cell viability at control levels. In contrast, neither NAC nor Trolox exerted protective effects at the concentrations tested, and NAC pretreatment further exacerbated cell death following 3,4-DMMC exposure (*p* < 0.05).

To gain further insight into the redox-related mechanisms underlying these observations, ROS/RNS production was evaluated under the same experimental conditions. As shown in Figure 3B, AA, NAC, and Trolox all exhibited robust ROS-scavenging activity, significantly preventing the methylone- and 3,4-DMMC-induced increase in ROS/RNS levels (*p* < 0.0001) and maintaining them below control values.

Collectively, these findings indicate that oxidative stress is a key mechanism in the cardiotoxicity induced by methylone and 3,4-DMMC. However, antioxidant efficacy appears to be compound-specific: while all antioxidants were able to suppress ROS/RNS overproduction, only AA displayed cytoprotective effects, whereas NAC not only failed to confer protection but also potentiated toxicity, and Trolox showed no protective effect.

## 4. Discussion

This study provides new evidence that synthetic cathinones can exert direct cardiotoxic effects mediated by oxidative stress. The heart is inherently vulnerable to xenobiotic-induced injury due to its high metabolic demand, reliance on mitochondrial oxidative phosphorylation, and limited antioxidant capacity, rendering cardiomyocytes particularly sensitive to redox imbalance [24]. Here, we demonstrate for the first time that methylone and 3,4-DMMC induce cardiomyocyte death in vitro. Notably, 3,4-DMMC exhibited markedly greater toxicity than methylone, a finding consistent with earlier reports of its higher cytotoxicity in neuronal [25] and hepatic [26] models. For both compounds, cell death was concentration-dependent, while no significant differences were observed between 24 h and 48 h exposures, suggesting that most cellular damage occurs shortly after exposure. The rapid onset of cell death further underscores the potential relevance of these findings for acute cardiac events associated with synthetic SC use.

The decline in cell viability assessed by the MTT assay reflects underlying mitochondrial dysfunction. Concurrently, methylone and 3,4-DMMC rapidly increased intracellular ROS and RNS levels within hours of exposure, indicating that oxidative stress is an early event rather than a downstream consequence of mitochondrial damage. Excessive ROS accumulation can then initiate opening of the mitochondrial permeability transition pore (mPTP), leading to cytochrome c release, caspase activation, and subsequent cell death through apoptosis or necrosis [27,28]. The co-occurrence of mitochondrial impairment and sustained ROS/RNS overproduction suggests a synergistic mechanism driving cardiomyocyte toxicity, underscoring the pivotal role of redox imbalance in this process. These findings align with an earlier work on another SC, mephedrone, which similarly induced mitochondrial dysfunction and oxidative stress in isolated rat heart mitochondria [18].

Although studies specifically addressing SC toxicity in cardiac cell models remain limited, their cytotoxic effects are well documented in other systems, including hepatocytes [26,29,30], neuronal cells [31,32,33,34], renal cells [23], and in vivo models [35,36,37]. The consistency of these responses across diverse cell types reinforces the notion of shared mechanistic pathways among SCs. In addition to ROS induction, previous studies have reported disrupted mitochondrial dynamics, loss of membrane potential (∆Ψm), and activation of apoptotic and, in some cases, autophagic and necrotic pathways. These observations suggest that oxidative stress may act as an upstream trigger for multiple cell death pathways, particularly apoptosis, following SC exposure.

In this context, our results identify oxidative stress and mitochondrial dysfunction as key contributors to methylone- and 3,4-DMMC-induced cardiotoxicity, although the exact nature of the ensuing cell death remains to be clarified. Future studies should delineate the downstream pathways activated by ROS overproduction in cardiomyocytes, including caspase-dependent and -independent apoptosis, autophagy, and necroptosis. Taken together, these findings support oxidative stress as a unifying mechanism of SC-induced toxicity, including in cardiomyocytes, and emphasize the need to extend these investigations to in vivo models to capture systemic cardiovascular effects.

Mechanistic insights were further supported by antioxidant experiments in which H9c2 cells were pretreated with three well-characterized antioxidants with distinct modes of action: AA, a direct ROS scavenger [38]; NAC, a thiol compound that acts both as a precursor of reduced glutathione and as a direct radical scavenger [39]; and Trolox, a water-soluble analogue of vitamin E that acts at the lipid membrane level [40]. All three antioxidants significantly suppressed methylone- and 3,4-DMMC-induced ROS/RNS generation, confirming their effective radical-scavenging capacity under our experimental conditions. However, only AA fully preserved cell viability, while NAC and Trolox, despite mitigating ROS/RNS accumulation, failed to protect against cytotoxicity. These results indicate that although oxidative stress plays a central role in SC-induced cardiotoxicity, successful protection also depends on the antioxidant’s ability to maintain physiological redox signaling and target the relevant subcellular compartment.

The ability of AA to fully prevent cytotoxicity reinforces oxidative stress as a major driver of methylone- and 3,4-DMMC-induced cardiac injury. This finding highlights the importance of water-soluble antioxidants in neutralizing intracellular ROS and agrees with previous studies demonstrating AA-mediated protection against oxidative agents in cardiac cells [41,42,43,44]. In contrast, NAC, while reducing ROS levels, failed to provide protection and even reduced cell viability when administered alone. This was unexpected, given that previous studies consistently reported a protective role for NAC (within the same concentration range), including against SC toxicity in neuronal SH-SY5Y cells [33] and primary rat hepatocytes [30]. Nevertheless, the increase in cell death observed here can be reasonably explained by the paradoxical role of ROS in cellular physiology: at low to moderate levels, ROS are essential for signaling pathways that regulate survival and adaptation, whereas at high levels they cause oxidative damage to DNA, proteins, and lipids [45]. Excessive NAC may shift the intracellular environment toward an over-reduced state, disrupting ROS-dependent signaling and triggering apoptosis or necrosis, as reported in earlier studies [46]. This interpretation is further supported by recent evidence in human HK-2 renal cells exposed to methylone and 3,4-DMMC, where NAC potentiated toxicity by interfering with autophagy-dependent survival responses and thereby enhancing apoptosis [23].

Unlike NAC, Trolox did not compromise cell viability but similarly failed to confer protection. A plausible explanation is that the primary sites of ROS/RNS generation by methylone and 3,4-DMMC are cytosolic and mitochondrial, where the membrane-targeted antioxidant activity of Trolox is unlikely to mitigate toxicity. Taken together, these divergent antioxidant responses underscore the complexity of redox modulation as a therapeutic strategy and highlight that not all ROS-scavenging strategies confer cytoprotection. Effective antioxidant intervention likely requires targeting the appropriate subcellular compartment while preserving physiological redox signaling. In this context, AA emerges as the most promising candidate for mitigating SC-induced cardiac injury due to its ability to neutralize intracellular ROS without disrupting essential redox-dependent processes.

While our findings strengthen the mechanistic link between oxidative stress and SC-induced cardiac injury, certain limitations must be acknowledged. First, although the SC concentrations tested fall within the range commonly used in in vitro studies [23,29,31,34], they are considerably higher than the micromolar levels typically detected in intoxication cases [11,12,47]. However, it is important to note that SCs are frequently consumed in polydrug settings, where pharmacological interactions can lower toxicity thresholds [48,49,50,51]. Consistent with this, mixtures of methylone and MDPV in rats produced synergistic cardiovascular effects [52]. Second, H9c2 cell line cannot fully recapitulate the complexity of human cardiac physiology. Future work should incorporate human stem cell-derived cardiomyocytes and appropriate in vivo models to validate these mechanisms in a more physiologically relevant context. Finally, only a limited set of antioxidants was tested. Broader evaluation, particularly of mitochondria-targeted antioxidants, may yield more effective strategies to counteract SC-induced dysfunction compared with general ROS scavengers.

## 5. Conclusions

This study demonstrates that the SCs methylone and 3,4-DMMC exert direct cardiotoxic effects in H9c2 cardiomyocytes, with 3,4-DMMC showing greater potency. Oxidative stress and mitochondrial dysfunction emerge as key mediators of toxicity, with rapid and sustained ROS/RNS accumulation driving cellular injury, expanding the current knowledge of SC-induced cardiotoxicity beyond sympathetic overstimulation. All tested antioxidants efficiently reduced ROS/RNS overproduction; however, only AA restored cell viability, while NAC and Trolox, despite their ROS-scavenging activity, failed to prevent cytotoxicity. These divergent outcomes indicate that antioxidant efficacy depends not only on the ability to neutralize ROS but also on preserving redox signaling and reaching the relevant intracellular compartments. AA’s effectiveness likely stems from its balanced redox-modulating capacity and ability to counteract both cytosolic and mitochondrial oxidative stress. Given its established safety profile and clinical availability, AA may represent a promising adjuvant strategy to mitigate acute cardiac oxidative stress induced by SCs. Nevertheless, confirmation in in vivo and clinical models is required to establish its therapeutic relevance and optimal dosage.

## Data Availability

The original contributions presented in this study are included in the article. Further inquiries can be directed to the corresponding author.

## Supplementary Materials

The following supporting information can be downloaded at: https://www.mdpi.com/article/10.3390/toxics13110998/s1, Figure S1: Chemical structures of methylone and 3,4-DMMC; Figure S2: Nonlinear regression models for the cell death induced by (A) methylone and (B) 3,4-DMMC in H9c2 cells, as evaluated by the MTT assay after 24 h and 48 h of exposure. The mean effects were fitted to the logit function. Dotted lines represent the 95% confidence band of each fit. Results were obtained from three independent experiments, performed in duplicate. Embedded tables: estimated IC_50_ values for each compound at the respective exposure time and *p* value for group comparison (24 vs. 48 h) of global fits.

## Abbreviations

The following abbreviations are used in this manuscript:

3,4-DMMC	3,4-Dimethylmethcathinone
AA	Ascorbic acid
DCFH-DA	2,7-Dichlorodihydrofluorescein diacetate
DMSO	Dimethyl sulfoxide
EUDA	European Drug Agency
FBS	Fetal bovine serum
IC_10_/IC_40_/IC_50_	Concentrations inhibiting 10%, 40% or 50% of cell viability, respectively
MDMA	3,4-Methylenedioxymethamphetamine
MDPV	3,4-Methylenedioxypyrovalerone
mPTP	Mitochondrial permeability transition pore
MTT	3-(4,5-Dimethylthiazol-2-yl)-2,5-diphenyltetrazolium bromide
NAC	N-Acetylcysteine
NPS	New psychoactive substance
RNS	Reactive nitrogen species
ROS	Reactive oxygen species
SCs	Synthetic cathinones
SEM	Standard error of the mean
