# Supplementary material for: From Euphoria to Cardiac Stress: Role of Oxidative Stress on the Cardiotoxicity of Methylone and 3,4-DMMC
Source: Toxics. 2025 Nov 20;13(11):998. doi: 10.3390/toxics13110998 (PMC12656588; doi:10.3390/toxics13110998)
Supplement: Supplementary file 1 [file toxics-13-00998-s001.zip › toxics-3936450-supplementary.pdf]

SUPPLEMENTARY DATA

From Euphoria to Cardiac Stress: Role of Oxidative Stress on the Cardiotoxicity of Methylone and 3,4-DMMC

Maria Moreira [1,†], Verónica Rocha [1,†], Ana Margarida Araújo [2,*] and Márcia Carvalho [1,2,3,*]

1 Instituto de Investigação, Inovação e Desenvolvimento Fernando Pessoa (FP-I3ID), Fernando Pessoa University, Fernando Pessoa Teaching and Culture Foundation, Praça de 9 de Abril 349, 4249-004 Porto, Portugal

2 Laboratório Associado para a Química Verde/Rede de Química e Tecnologia (LAQV/REQUIMTE), Laboratory of Bromatology and Hydrology, Department of Chemical Sciences, Faculty of Pharmacy, University of Porto, 4050-313 Porto, Portugal

3 RISE-Health, Faculty of Health Sciences, Fernando Pessoa University, Fernando Pessoa Teaching and Culture Foundation, Rua Carlos da Maia 296, 4200-150 Porto, Portugal

* Correspondence: amaraujo@ff.up.pt (A.M.A.); mcarv@ufp.edu.pt (M.C.)

† These authors contributed equally to this work.

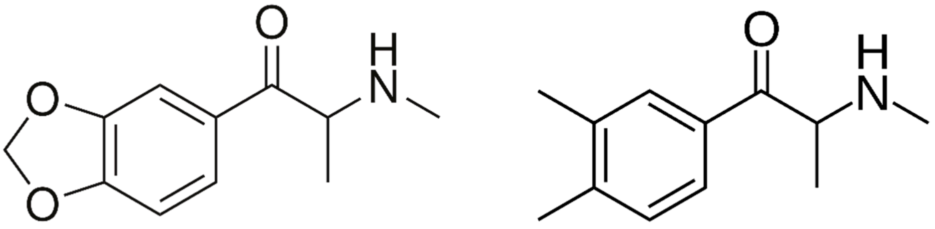

Methylone   3,4-DMMC

Figure S1. Chemical structures of methylone and 3,4-DMMC

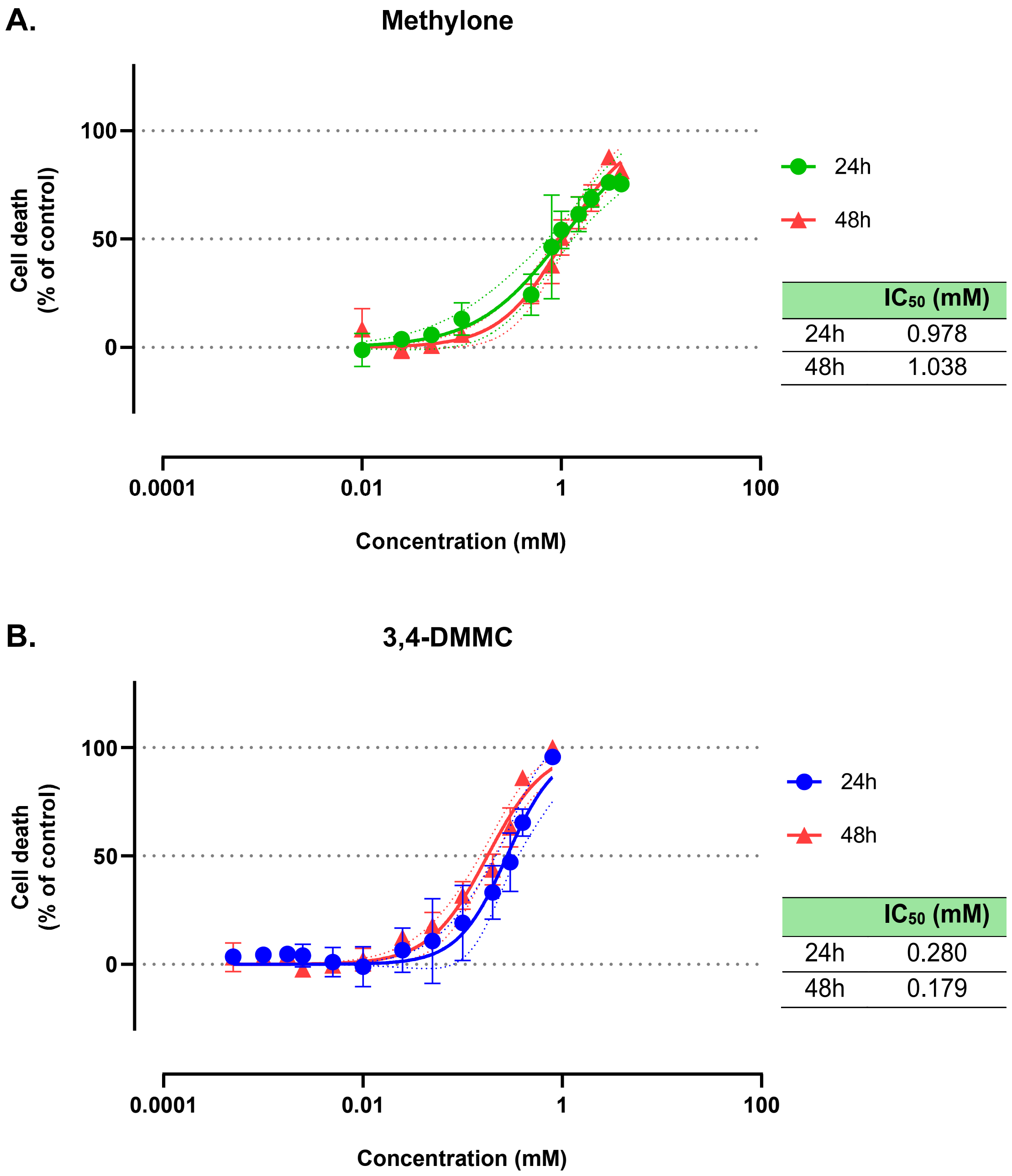

	IC_{50} (mM)
24h	0.978
48h	1.038

	IC_{50} (mM)
24h	0.280
48h	0.179

Figure S2. Nonlinear regression models for the cell death induced by (A) methylone and (B) 3,4-DMMC in H9c2 cells, as evaluated by the MTT assay after 24 h and 48 h of exposure. The mean effects were fitted to the logit function. Dotted lines represent the 95% confidence band of each fit. Results were obtained from three independent experiments, performed in duplicate. Embedded tables: estimated IC50 values for

each compound at the respective exposure time and p value for group comparison (24 vs. 48 h) of global fits.
